# Evaluating priority setting success in healthcare: a pilot study

**DOI:** 10.1186/1472-6963-10-131

**Published:** 2010-05-19

**Authors:** Shannon L Sibbald, Jennifer L Gibson, Peter A Singer, Ross Upshur, Douglas K Martin

**Affiliations:** 1 School of Health Studies The University of Western Ontario London, Ontario, N6A 5B9, Canada; 2 University of Toronto Joint Centre for Bioethics 88 College Street Toronto, Ontario, M5G 1L4, Canada; 3 McLaughlin-Rotman Centre for Global Health, University Health Network and University of Toronto Faculty of Medicine MaRS Centre, South Tower 101 College Street, Suite 406 Toronto, Ontario, M5G 1L7, Canada; 4 University of Toronto Joint Centre for Bioethics & University of Toronto School of Public Health Sunnybrook Health Science Centre 88 College Street Toronto, Ontario, M5G 1L4, Canada; 5 University of Toronto Joint Centre for Bioethics & University of Toronto Department of Health Policy, Management and Evaluation, 88 College Street Toronto, Ontario, M5G 1L4, Canada

## Abstract

**Background:**

In healthcare today, decisions are made in the face of serious resource constraints. Healthcare managers are struggling to provide high quality care, manage resources effectively, and meet changing patient needs. Healthcare managers who are constantly making difficult resource decisions desire a way to improve their priority setting processes. Despite the wealth of existing priority setting literature (for example, program budgeting and marginal analysis, accountability for reasonableness, the 'describe-evaluate-improve' strategy) there are still no tools to evaluate how healthcare resources are prioritised. This paper describes the development and piloting of a process to evaluate priority setting in health institutions. The evaluation process was designed to examine the procedural and substantive dimensions of priority setting using a multi-methods approach, including a staff survey, decision-maker interviews, and document analysis.

**Methods:**

The evaluation process was piloted in a mid-size community hospital in Ontario, Canada while its leaders worked through their annual budgeting process. Both qualitative and quantitative methods were used to analyze the data.

**Results:**

The evaluation process was both applicable to the context and it captured the budgeting process. In general, the pilot test provided support for our evaluation process and our definition of success, (i.e., our conceptual framework).

**Conclusions:**

The purpose of the evaluation process is to provide a simple, practical way for an organization to better understand what it means to achieve success in its priority setting activities and identify areas for improvement. In order for the process to be used by healthcare managers today, modification and contextualization of the process are anticipated. As the evaluation process is applied in more health care organizations or applied repeatedly in an organization, it may become more streamlined.

## Background

Priority setting is a challenge for all health systems because demand for health care usually exceeds available resources. Decision-makers struggle with determining how resources should be used to provide high quality patient care services in a sustainable way. In recent years, in Canada and elsewhere, there has been an increasing level of scrutiny regarding how these decisions are made. Both consumers and funders are demanding greater accountability for how limited health resources are used to meet health system goals. Considerable progress has been made in the last decade on developing theoretical frameworks and practical strategies to guide and evaluate priority setting [1]. However, there remains no consensus regarding which, or whose, values should guide these decisions and how these values should inform priority setting decisions.

Healthcare decision makers in publicly funded systems are under growing pressure to improve their priority setting processes and to be more accountable for their decisions. This problem persists in both the developed and the developing world throughout various health care systems and organizations. As a global concern the determination of best practices in priority setting is internationally significant. Decision makers may find some guidance on making difficult resource decisions from economic and/or ethical principles [2-4], or they may learn from national and international descriptions priority setting activities [5-8]. However, despite current efforts to create a comprehensive approach to priority setting (see for example [9,10] etc.), there remains no single tool that can help evaluate, and therefore guide, priority setting [11].

An important first step to evaluating priority setting is knowing what good, or successful, priority setting looks like [12]. A definition of success can be used to shape practice in health care priority setting. In a previous study we developed a conceptual framework that aimed to define successful priority setting informed by a multi-stakeholder perspective (decision/policy-makers, scholars and patients) (see Table 1 and Table 2) [13]. The framework was developed based on three empirical studies, each using different qualitative methods, which provided a perspective on key elements necessary for successful priority setting.

**Table 1:** Conceptual Framework

	Elements
**PROCESS**	Stakeholder Engagement
	
	Explicit Process
	
	Clear And Transparent Information Management
	
	Consideration Of Values and Context
	
	Revision Or Appeals Mechanism

**OUTCOMES**	Stakeholder Understanding
	
	Shifted Resources
	
	Decision Making Quality
	
	Stakeholder Acceptance & Satisfaction
	
	Positive Externalities

**Table 2:** Description of Elements in the Conceptual Framework

PROCESS CONCEPTS 1. Stakeholder Engagement Stakeholder engagement refers to an organization's efforts to identify the relevant internal and external stakeholders and to involve those stakeholders effectively in the decision-making process. This should include, at a minimum, administrators, clinicians, members of the public and patients. To ensure adequate engagement, identifying and engaging stakeholders should involve multiple techniques, such as round tables, open forums, departmental meetings. There should be a genuine commitment from the organization to engage stakeholders effectively through partnership and empowerment. Stakeholder engagement is also concerned with stakeholder satisfaction regarding the level of their involvement in the decision-making process.
2. Explicit Process An explicit process is one that is transparent, not only to decision makers, but also to other stakeholders. Adhering to a predetermined process can enhance trust and confidence in the process. Transparency means knowing who is making the decision as well as how and why the decision will be made. Communication needs to be well coordinated, systematic and well-planned. All stakeholders (internal and external) should be probed for information relevant to the priority setting decisions, and information should be communicated effectively using multiple vehicles (town-hall, departmental meetings, memos, emails, etc.)
3. Clear and Transparent Information Management Information management refers first to the information made available to decision makers during the priority setting process. This includes what was used and what was perceived to be lacking. Second, information management considers how the information was managed, including how it was collected and collated. Relevant information includes, but is not restricted to: health outcomes data, economic data (such as cost effectiveness analyses), community needs assessment, current policies or policy reports, and the experiences of both clinicians and patients.
4. Consideration of Values and Context Values and context are important considerations in any priority setting process, including the values of the organization, the values of staff within that organization, and the values of other stakeholders (such as patients, policy makers, politicians, and members of the community). The mission, vision and values of the organization should guide priority setting. Priority setting decisions should be based on reasons that are grounded in clear value choices, and those reasons should be made explicit. This also involves not only looking within the organization at previous priority setting decisions, but also studying what other health care organizations are doing. This would involve looking at organizations in the local community, at other health care organizations with similar mandates, as well as looking at the other levels of health care provision. Context is distinct from values and considers the organization's goals in the health care environment, as articulated in its strategic directions.
5. Revision or Appeals Mechanism A revision process is a formal mechanism for the review of decisions, and for addressing disagreements constructively. Such a mechanism is important to ensure the priority setting process rules and requirements are communicated clearly ahead of time. The dual purposes of a revision process are to: 1) improve the quality of decisions by providing opportunities for new information to be brought forward, errors to be corrected, and failures in due process to be remedied; and 2) to operationalize the key ethical concept of responsiveness.

OUTCOME CONCEPTS 1. Stakeholder Understanding Stakeholder understanding implies more than basic knowledge of the process. It assumes stakeholders have gained insight into the priority setting process (e.g., its goals, rationale and rationale for its decisions) and/or the organization (e.g., mission, vision, values, and strategic plan). As stakeholder understanding increases, stakeholder acceptance and confidence should also increase.
2. Shifted Resources A successful priority setting process results in the allocation of budgets across portfolios, changes in utilization of physical resources (e.g., operating theatre schedules, bed allocations) or possibly changes in strategic directions. Effort that does not result in change may encourage the perception among stakeholders that the process is an inefficient use of time or is done for the outward appearance ('window-dressing') of pre-determined outcomes. A reaffirmation of previous resource allocation decisions (e.g. the previous year's budget) may, in some circumstances, be seen as a success.
3. Decision Making Quality Decision making quality relates to appropriate use of available evidence, consistency of reasoning, institutionalization of the priority setting process, alignment with the goals of the process, and compliance with the prescribed process. It also captures the extent to which the institution is learning from its experience in order to facilitate ongoing improvement. This component is most visible as subsequent iterations of priority setting are evaluated; where consistency and building on previous priority setting would be indicative of a successful process. Institutional learning, increased institutionalization of priorities, more efficient decision making, more consistent decision making, and increased compliance with decisions (i.e. 'buy-in') are all valuable outcomes of successful priority setting that are difficult to achieve. Institutional learning from experience facilitates ongoing institutional improvement, which is made more visible as subsequent iterations of priority setting are evaluated.
4. Stakeholder Acceptance and Satisfaction It is important to consider the satisfaction of all stakeholder groups, both internal and external to the hospital (community groups/public and governmental health agencies/ministries of health). Successful priority setting leads to increased satisfaction over multiple decision cycles. Stakeholder acceptance is indicated by continued willingness to participate in the process (i.e. 'buy-in') as well as the degree of contentment with the process. Stakeholders may be able to accept priority setting decisions, even if they may not always agree with the outcomes.
5. Positive Externalities Positive externalities can act as a sort of check and balance, ensuring information is made transparent to stakeholders through various avenues, and/or establishing good practices for budgeting in other health care organizations. As an indicator of success, externalities may include positive media coverage (which can contribute to public dialogue, social learning, and improved decision making in subsequent iterations of priority setting), peer-emulation or health sector recognition (e.g. by other health care organizations, CCHSA, etc), changes in policies, and, potentially, changes to legislations or practice.

Evaluating success in priority setting has been difficult since there is little agreement on a definition of priority setting success. The evaluation of success is distinct from other discipline-specific evaluation strategies, for example, fairness ('accountability for reasonableness'[14]), or evaluation of value for money [15-17]. Several studies have presented ideas for evaluating success in priority setting including: economic evaluations [18,19], checklists incorporating both pragmatic and ethical ideas [10], criteria-based framework (objectives and context, methodology, process issues, and study outcomes) [20], outputs-based measures (usefulness re-allocation, improved patient outcomes) [21], and an ethical standards model (integrating patients health, expertise, unmet health needs, and benefit to community) [22]. These ideas are important for understanding and conceptualizing success in priority setting, however, alone they do not provide clear guidance.

Gibson et al [2], described what were termed "parameters" of success (organizational priorities, staff and community, efficiency and fairness) however their participant group was not inclusive (only senior managers/board members) and might not represent the views of non-senior staff. Teng et al. [23] described "essential elements to improve priority setting". Their study found that decision makers desired a more explicit framework or process for priority setting; however, their elements do not provide a complete illustration of success in priority setting and further the authors also indicated that lack of tools for priority setting is a barrier to improvement - - the conceptual framework and evaluation process developed in this paper fills this gap [23]. Other studies focus exclusively on process - which is important but not sufficient for determining the success of priority setting [24]. Taken together, these studies contribute necessary elements to our understanding of evaluating successful priority setting but alone are insufficient as they do not provide a comprehensive (multi-faceted) evaluation process to do so.

Evaluating success in priority setting - indeed in any area - is difficult to do when 'success' has not been defined. Outside of priority setting, success has been defined and measured. For example, in education, success has been measured using concepts such as creativity, fluency, originality, and elaboration [25] in business, 'corporate success' has been defined [26] and many authors have explained how to achieve 'business success' (for example: [27]). Within the priority setting literature, the few studies that have examined pieces of successful priority setting [4,10,21,28] are not in agreement on underlying assumptions (i.e. underlying values) and often conflict.

An evaluation process is needed that will a) provide concrete guidance, b) help identify specific opportunities to improve decision making and c) show if the allocation of resources has improved. This process should be comprehensive and evidence-informed. In this paper we aim to show why existing evaluation approaches are insufficient, and present an evaluation process to evaluate success in priority setting.

A comprehensive evaluation process is one that would integrate (and support) what is known from existing literature and potentially introduce new, important, elements that have not been established in the literature. We created an evaluation process that can be used by healthcare managers to evaluate the success of their priority setting processes based on the ten elements from our conceptual framework. The evaluation process aims to capture a broad overview of a complex phenomenon, or a global index [29] (see Table 3), that can evaluate a priority setting initiative and help identify strengths and opportunities for improvement. To examine its validity and usability we conducted a pilot test in a mid-size community hospital in Ontario. In this paper we identify indicators of success for evaluating priority setting processes (the evaluation process), and present the results of a pilot study which tested whether this evaluation process was effective in evaluating priority setting success.

**Table 3:** Scale Development - A Global Index

(The following information is taken from 'Clinimetrics' by AR Feinstein (Feinstein, 1987))

Feinstein uses the term 'global' to refer to content which is a broad overview of a complex phenomenon. (p. 92)

"When we form a composite index or a global scale for a complex phenomenon, the scientific goal is to get an overall appraisal of the total phenomenon, not to preserve the identity of each component. If we want to know about each component, we would use or review separate indexes for the component." (p.100)

The main disadvantage of a global index is that the results are often not replicable by other observers (inter-rater reliability; reproducible consistency). However, global indexes are valuable in denoting changes of state - that is, individual ratings using the same scale will be reasonably well standardized (internal validity).

Global indices can have a high intra-rater consistency (when the same person applies it more than once, there will be standardization) but often a low inter-rater consistency (when applied by separate researchers). Since global indices permit measuring states of change, comparable results can be achieved. Further, it is possible to acquire validity in measuring since measuring change or transition ratings often yields consistency because raters are likely to use similar criteria when measuring, for example: "better, no change, worse".

Feinstein argues that "a collection of transition ratings may be reasonably well standardized within and among the individual members of the group" (p. 97). That is, if the evaluation tool created in this thesis were used to evaluate the achievement of success in priority setting in one organization, it would be possible to evaluate states of change, or to evaluate improvement.

## Methods

There are two distinct phases in this study: (1) the creation of the evaluation process, and (2) assessing the validity, usefulness, and applicability of the evaluation process.

### (1) Creation of the evaluation process

The creation of the evaluation process was a multi-step iterative process which flowed from the ten elements of the conceptual framework (for a detailed discussion on the formulation of the framework please see Sibbald et al., 2009 [13]). The first step in its development was to pose questions that attempted to operationalize each element of the conceptual framework, and that mapped onto the ethical and practical goals of priority setting. Both quantitative and qualitative questions that related to procedural and substantive dimensions of priority setting were used. We then mapped these questions out across three specific evaluation processes: a survey, interviews, and document analysis. In the third step we revised our questions based on feedback we received from stakeholders. This last step was an iterative process of proposing evaluation indicators and refining them based on the feedback received from stakeholders. The final evaluation process was further revised through face and content validity testing, and also 'usability' based on empirical application (pilot test).

Our survey, interviews, and document analysis constitute the methods of the evaluation process to identify success in priority setting. The survey consisted of 35 questions, the interview guide outlines six overarching questions (with various probes), and there were 13 questions in the document analysis guide (additional file 1 contains a complete original versions of evaluation process).

### (2) Assessing the validity, usability, and applicability of the evaluation process

The validity of the evaluation process was tested in two steps. First, face and content validity of the survey, interview guide, and document analysis were tested by circulating them to an interdisciplinary group of researchers and decision/policy makers (a face content validity (FCV) panel). The FCV panel assessed the readability, clarity, and how well the questions captured (content validity) or reflected (face validity) the ten elements of the conceptual framework. In total, 12 individuals made up the FCV panel including four priority setting scholars and eight decision makers involved in priority setting decisions (Table 4). Four out of the seven priority setting scholars were also clinicians involved in priority setting decisions; this group represented both 'experts' and 'users'.

**Table 4:** Face & Content Validity Participants. (PS = Priority Setting)

	Category	Nationality
1	PS scholar	Canada

2	PS scholar	Uganda

3	PS scholar	Zimbabwe

4	PS scholar	United States

7	Policy Maker	Canada

8	Decision Maker	Canada

9	Decision Maker	Canada

10	Decision Maker	Canada

11	Decision Maker	Canada

12	Decision Maker	Canada

13	Decision Maker	Canada

14	Decision Maker	Canada

The second step (the focus of this paper) tested the real-world applicability and usability of the evaluation process by administering it in a mid-size urban hospital (a pilot test). The pilot test consisted of implementing the survey, conducting interviews with the developed question guide, and completing document analysis with our tool in a health organization, and then getting feedback from the organization on the results as well as the implementation process [30].

#### Setting

The pilot test was conducted in a mid-sized acute care urban community hospital in Ontario. The hospital is situated in a high-growth area, has approximately 2000 staff and approximately 300 beds, and provides a comprehensive range of acute hospital-based services, including a large child and maternal health program, critical care, etc., and complex continuing care. The hospital was selected because of their interest in priority setting activities and their willingness to support bioethics research. At the time of the pilot study, the hospital had recently completed a substantial budget allocation process, which provided a good opportunity for a retrospective evaluation. We sought to evaluate the hospital's 2007/08 budgetary process, which was conducted over a 4-week period in the summer of 2006. The results we present are largely descriptive of this priority setting (budgeting) process.

#### Sampling and Participants

Participants in the pilot test study were employees of the hospital and included those who were directly involved (senior management, administration, program managers and directors) and indirectly involved (e.g., front line nurses and physicians, ancillary staff etc.) in the 2007/08 budgeting process. Sample size was not formally calculated; the goal of implementing the evaluation process was not to achieve saturation or generalization but rather to learn from individuals who had participated in the budgeting process, and test the functionality of the evaluation process in a real-world setting.

Participants for the survey were recruited via internal email, to all hospital employees with an email account (n = 2000). In total, 105 hospital employees responded to the online survey, however 27 surveys were not analyzed because they were incomplete (n = 78; Table 5).

**Table 5:** Survey Respondents

Job Title	
Front Line Staff	40

Program Directors	13

Program Managers	8

Senior Leadership Team	1

Other/did not say	16

**TOTAL**	**78**

'Front line' was used to define health care professionals who work at the bedside and have direct contact with patients (nurses, allied health, and physicians). 'Other' captured hospital employees such as clerical and engineering staff.

Sampling of interview participants was done first using a convenience sampling (availability) and then a combination of theoretical sampling (people who were involved in a significant aspect of the priority setting initiative) and snowball sampling (asking participants to refer us to others). Twenty hospital managers (program managers, directors, and senior leadership) were invited to participate in a one-on-one interview and nine of them participated (Table 6).

**Table 6:** Interview Participants

Position	
Program Directors	4

Senior Leadership Team	1

Program Managers	3

Other	1

**TOTAL**	**9**

In total, 18 documents were analyzed (e.g. strategic plan, budgetary information, meeting minutes and memos, as well as presentations and email communication): 10 documents were collected from the department responsible for 'decision support' (i.e., providing data, technical, administrative and procedural support), 4 from the hospitals internal website, and two through email communications with Senior Leadership; other documents were obtained directly from the Senior Leadership Team (Table 7).

**Table 7:** Documents Analyzed

Documents	
Decision Support documents	10

Website information	4

Email communications	2

Meeting information	2

**TOTAL**	18

### Data Collection and Analysis

#### i. Face and Content Validity Panel

Data collection for the FCV panel took place from April-May 2007. All three components of the draft evaluation process were disseminated to participants via email. Panellists were provided with the conceptual framework (the ten elements) and a worksheet with all of the questions divided into their format (surveys, interviews, document analysis). Panellists were asked to comment on the face and content validity of the survey, interview guide, and document analysis guide, i.e., do the survey, interview guide, and document analysis guide reflect the domains of successful priority setting listed in the framework? Comments were read and analyzed independently, and then analyzed in aggregate to reach consensus amongst panellists. The data gathered was used to refine the survey, interview guide, and document analysis guide.

#### ii. Pilot Test

The pilot test was conducted in May-July 2007. A link to the on-line survey was sent through email; the response rate of the survey could not determined (there are over 2000 employees at the hospital, and while most have an organization account, not all have activated, or used, them). Key informant interviews were used to validate information collected by the survey and to gather individual experiences and perspectives. An interview guide was used, and conversations were audio-taped and transcribed. Document analysis (reviewing annual reports, strategic plans, and meeting minutes, etc.) provided both qualitative and quantitative data along with insight into the budgeting process and outcomes; collection and analysis were on-going throughout the survey and the interviews.

Data analysis of the pilot test proceeded in two steps. In the first step, results from each portion of the evaluation process (surveys, interviews, document analysis) were analyzed independently: survey data were analyzed using simple descriptive statistics and modified thematic analysis for the open-ended questions; interviews and the documents were analyzed using thematic coding, guided by the ten elements in the conceptual framework. Data was then synthesized and re-analyzed both 'within' and 'between' material to discover common themes. Based on the data analysis, the research team developed recommendations for future priority setting activities. The recommendations and description of the process were presented in an eight-page report to senior management. This report was the input into a debriefing session (second round of interviews) in order to determine the usefulness of the results.

In the second step of data analysis the evaluation process was analyzed. This was done using feedback received from three debriefing interviews (one with the CEO, one with the VP of Finance (CFO) and one with the Leader of Organizational Development and Ombudsperson) as well as through researcher experience.

Validity of our findings was maintained throughout the study firstly by regularly presenting data and analysis to a group of interdisciplinary researchers to ensure accuracy and lack of personal bias. This interdisciplinary analysis was a valuable part of data analysis allowing any differences to be discussed and resolved through ongoing discussion. Second, all research activities were rigorously documented by the researcher to permit a critical appraisal of the methods [31]. Third, our debriefing interviews acted as a form of member check to ensure our findings were reasonable.

### Research Ethics

Research ethics approval was obtained through both the University Review Office and the Hospital Research Ethics Board at the pilot hospital, which requested not to be identified in this paper. Informed consent was obtained from each participant. All raw data was (and is) protected as confidential and is available only to the research team. No individuals were identified in dissemination without explicit agreement.

## Results

### (1) Pilot test results

In total, 78 hospital employees completed the survey while 27 started the survey but did not complete it; 9 senior management hospital employees also participated in interviews. The results from the survey, the interviews, and the document analysis are presented in aggregate in this section under each of the heading of the conceptual framework. While 78 surveys were collected and analyzed, fewer than 78 respondents answered each question and therefore data presented will reflect only the number of respondents who replied to each question (a summary of the closed survey results is available in additional file 2).

### Process Components

#### Stakeholder Engagement

The survey contained seven questions pertaining to stakeholder engagement. Fifty-nine per cent (n = 46) of respondents stated they were 'not at all involved' in the budgeting process, 21.8% (n = 17) were 'very involved' and 19.2% (n = 15) were 'somewhat involved'. This question was followed by a question on the satisfaction of involvement: 37.3% (n = 28) were not satisfied with their involvement, 26.7% (n = 20) were satisfied, and the remainder (36%) were not sure. When level of involvement is compared with satisfaction 53% (n = 17) of those who somewhat or very involved were satisfied with their involvement, whereas only 6.5% (n = 3) of those who not involved were satisfied with their involvement, 41% (n = 19) were satisfied and the majority (52.5%; n = 24) did not know, or did not respond. Respondents had a chance to explain their answer in an open-ended question. Twenty respondents commented that there was not enough involvement or input from front line staff. Many participants (both in interviews and the survey) pointed to tight timelines as a primary reason for lack of broader consultation. 26% (n = 20) thought that other staff/employees should have been involved in the process (more front-line, unions, and allied health professionals).

A key strength of the 2007/08 budgeting process was the involvement of the program director and managers, which was a significant departure from past budgeting exercises that were largely driven by senior management decision-making alone. Managers (including senior leadership team (SLT), program directors, and program managers) were the group most involved in the budgeting process: 90.9% (n = 20) of managers who completed the survey reported being somewhat or very involved in the budgeting process. By contrast, front line staff was least involved in the budgeting process: 87.5% (n = 35) of front line staff who completed the survey reported not being involved in the budgeting process at all.

Interview participants all agreed that front line staff should have been more involved, and that increased consultation and engagement of external stakeholders, such as community groups, the public, and other health care providers, was required. Interviewees expressed an interest in greater internal collaboration on budgets to capture significant cross-departmental interdependencies as well as more communication throughout the process, especially in the form of inter-departmentally, where there seemed to be a lack of information sharing.

They didn't seem to get their groups (front line) involved. The other thing I think was a struggle was getting the programs talking to each other and some of the clinical areas engaged as to where the pushing factors were in the organization and what decisions were made and how they might impact on the other areas. (Senior Leader)

In survey questions about methods of engaging stakeholders, 68% (n = 53) of survey respondents did not know if there were multiple methods of stakeholder engagement, and 72.5%; n = 50 did not know if the methods were effective. Open end respondents said that there needed to be more opportunities for inter-departmental discussion.

Formal documentation or records of meetings about the 2007/08 budget (such as minutes) were limited as departmental budgetary discussions were mostly informal and records were unavailable or did not exist.

#### Explicit Process

Document analysis showed, and interview analysis confirmed, that the budget process followed an explicit and pre-determined timeline; however, participants complained that the time of year coupled with the short time frame impeded the rigour and transparency of the process. For example, participants expressed a lack of clarity in both the methods of decision making (50%; n = 39) of survey respondents did not know how decisions were made, and the individuals in charge of decision making (50%; n = 39) did not know who was making decisions. Interviewees were uncertain as to who was accountable for the final budget decisions (senior management team, the chief financial officer, or the provincial Ministry of Health):

Managers are maybe confused about whether they're making a decision within their own budgets or whether their director is or whether the senior team is ... the process is very iterative, it seems to go back and forth between levels. (Senior Leadership Team)

When asked if respondents knew who was making decisions, 37.2% (n = 29) said yes, 34.6% (n = 27) didn't know, and 28.2% (n = 22) said no. Respondents who answered yes were asked to specify who the decision makers were: 29 respondents offered a reply, most agreed that SLT had the decision making power, some thought that the board also had a hand in the decision making, and others felt that the decision making lay solely in the hands of the CFO. Interviewees were also uncertain as to who was accountable for the final budget decisions: the various options were the senior management team, the chief financial officer, and the MOH.

#### Information Management

There were three main inputs provided to decision makers during the budgeting process. First, information (such as previous budgets, funding structures, staffing information, etc) was managed largely through a pre-populated computer-based budgeting tool. Despite the tool helping to standardize the steps in the process, there were numerous frustrations around the functionality of the tool:

There were major hurdles because the template, the tool, was brand new and it had horrible hitches in it, bugs that should have been worked out, and the managers wasted a lot of time which was a crime and there was a lot of rework because it was brand new and it was done probably way too quickly. So they suffered ...that was a huge problem. (Senior Leadership Team)

Second, budgeting and expense information from nine 'peer' hospitals (those with similar demographics) was handed out to program directors/managers. Third, three decision making frameworks: the provincial Ministry of Health's framework (or 'Six Steps'), an ethical decision making framework, adapted from Gibson et al. [9], and an activity analysis tool developed at the hospital. The results showed these frameworks were rarely used, mostly due to insufficient information:

...trying to develop the operating budget which was a total frustration because there was no history ... or at least no accurate history as to how the previous budgets were developed.... You know, there was what did we spend historically in previous years but then the components that constituted the budget were not available so sort of a very frustrating time. (Director)

The four most common decision making inputs used by program directors/managers were: (1) capital need (e.g. equipment needs and/or updating existing materials), (2) interdependency (both intra- and inter-hospital impact), (3) strategic directions (including the hospital's mission, vision and values), and (4) other revenue sources (such as trust funds); none of which were included in the aforementioned provided inputs. Participants who were long-time employees of the hospital relied on their "own forecasting" and "personal knowledge" (or, tacit knowledge [32]) along with collegial relationships (internal and external) in their decision making.

In the survey, 61% (n = 36) thought that there should be other things considered in the budgeting process, the most common item being 'staffing levels', followed by population growth, under-funded areas, submissions to the provincial ministry of health, clinical priorities, and external factors (such as home care and family support set up).

#### Values and Context

The hospital had recently gone through a review of its strategic directions. The majority of survey respondents felt that the mission, vision and values of the hospital were considered in the 2007/08 budget (60%; n = 42); all interviewees felt that the budget followed the strategic directions, and saw at least some reflection of organizational values in the budget.

They were always reviewed - the mission, vision, values - were always reviewed at every budget session and the strategic directions, every budget had to be supported by the strategic directions (Senior Leadership Team)

Interviewees and survey respondents felt that staff values were not considered as much as they should have been. In interviews, participants related this to the emerging culture of shared accountability at the hospital.

I think it was a huge cultural shift for hospitals to start to be accountable and to start to be responsible for multi-year planning...And it's a whole paradigm shift. (Senior Leadership Team)

Several interviewees described how the new budgeting method and the resulting increased accountability would take time to adapt to and make happen.

While internal context appeared to play a role in the budgeting process (57.4%, n = 39 of survey respondents agreed), 78.3% (n = 65) of survey respondents did not know if there was integration of the hospital's 2007/08 budget with other health care organizations in the area. Interviewees discussed the shift towards the hospital budget aligning with the province's local health integration networks (LHINs), but interviewees were uncertain of the end-result of this shift or how it might affect the program's bottom line.

Respondents were asked about seven values and context items (mission, vision and values; strategic plan; context; culture; community values; patient values; and staff values) and their reflection in the outcome of the budget. The majority of respondents said that all elements were 'somewhat' or 'appropriately' reflected in the budget.

#### Revision Process

The 2007/08 hospital budget procedure did not have a formal revision process evident through all three methods of the evaluation process. What is more, most interviewees did not know what they would do if they wanted to contest a decision.

...if (program managers/directors) would have disagreed ... I guess, I made my proposal and there really isn't any place to go. (Program Leader)

Interviewees talked about the 'back and forth' that went on between different levels of management; however, these were seen largely as one-way discussions. Interviewees felt a two-way dialogue to allow changes to final budget decisions was lacking;

In the absence of (a way to appeal) I felt very frustrated that there really wasn't a second round ... a culture there where indeed that I could have a consultation where I had more of a chance to talk to a senior group. (Director)

Despite this, most interview participants indicated that they were sufficiently satisfied with the decision outcomes and that they would probably not access a revision process if one were available.

### Outcomes Components

#### Stakeholder Understanding

Sixty two per cent (n = 43) of respondents understood the outcome of the 2007/08 budget (either completely or somewhat). According to interview participants, the new budget process provided an opportunity for innovation in thinking and learning. Program directors and program managers had to learn the tool as well as the intricacies of budgeting. Interviewees felt that the priority setting process improved their understanding of the budget process, of spending in other areas of the hospital, and of the accountability required in the budget. Overall, interviewees felt the new budget process was a positive opportunity for learning, understanding, and innovation in thinking.

...the biggest outcome was that the managers learned what was in their budget...it was a huge learning curve it was a huge accountability piece too - - accountable for something that they built and they understood. ...and that's a new experience (Senior Director)

I think there's more understanding of what's in the budgets and I think there's more understanding of where the costs lie and what the impacts of some of the decisions that the programs are making on their budget. (Senior Leadership Team)

In order to get a sense of the learning that occurred during the 2007/08 budgeting process, respondents were asked to rank their familiarity with several items that may or may not have been considered during the budget: (1) mission, vision and values, (2) strategic plan; (3) context; (4) culture; (5) community values; (6) patient values; and (7) staff values; each of which were mentioned earlier in the survey surrounding information used in decision making. The majority of respondents did not become more familiar with any of the items.

#### Shifted Resources

When asked whether the 2007/08 budget process was consistent with previous budgets, the majority, 73.8% (n = 76) answered "I don't know", 9.7% (n = 10) felt it was consistent with previous budgets, and 16.5% (n = 17) said it was not. While most program directors and program managers welcomed the accountability and the flexibility to shift money within a department, some interviewees did not understand where re-allocated resources went or how funds were being used at an organizational level.

What's always useful... is to have the boundaries set ... don't ask me to get creative if I don't know how far I can go - I need to know how far I can go with this... I can't be real creative if I'm being cautious about money. (Senior Director)

This lack of transparency in reallocations was the cause of several participants' dissatisfaction with the overall process. Three survey participants said that they were not satisfied with the priority setting process due to small identifiable changes in the actual budget, stating it felt more like a "status-quo exercise". Although the complexity of budgeting material made it difficult to evaluate actual shifts or changes in resources on a hospital level, it became apparent through interviews that budgeting had led to resource shifts both within their own departments and between departments.

#### Decision Making Quality

According to those most involved in the budgeting process, the new approach to budgeting was an improvement in the quality of decision making. Since budgets from previous years had been set centrally by the finance department, many decision makers valued the increase in accountability. Interviewees felt that the changes increased their overall awareness of the organizational budget. Those who were involved in the process stated the computer-based budget tool was a source of frustration; others saw the new tool as an increase in accountability and as a "work in progress (that will) improve over time". (Program Director)

Senior Management encouraged decision makers to approach budget decision making not just from a mechanical stand-point, but also from a more "creative lens" (i.e. coming up with innovative solutions and not sticking to historical decisions). While some interviewees welcomed this approach, others were hesitant due to inadequate information and training/education. Participants felt that training before the budgeting process began could help to implement a creative approach to decision making and budgeting and would make them more comfortable in making priority setting decisions.

#### Stakeholder Acceptance

Participants generally accepted the budget (in the survey, 54.3%, n = 38 either completely or somewhat accepted the outcomes of the budget). Other respondents were dissatisfied with the outcomes because they felt that they were unaware or uninformed. While a few interviewees were unhappy with inter-departmental resource shifts, all interviewees seemed to accept the process.

Participants were asked how satisfied they were with the process behind the budget and were asked to explain their answer. Twenty-six respondents provided open-ended responses including: they were not satisfied because they did not know about the process, they were not involved in the process, or they were not engaged in the budget. Four respondents listed lack of, or poor, communication as a reason for decreased satisfaction.

External to the hospital, it was less clear whether there was acceptance and/or satisfaction with the budget process or outcomes. Once the budget was complete, it went back and forth to the MOH several times before agreement on its terms was reached. This was done at the upper management level and included little discussion with other stakeholders. Neither the public nor any community groups were directly involved in the budget process, making it difficult to get a sense of their acceptance and/or satisfaction.

#### Positive Externalities

Despite searching the media, and asking both survey and interview participants about information external to the organization, our evaluation found no evidence of 'positive externalities' (i.e., media reports, peer commentaries, or health sector responses) to suggest that others perceived the hospital's budgeting process to be successful.

##### Perceived Usefulness

The analysis of the pilot test was presented in an eight-page report organized according to the ten elements of the conceptual framework. Each section discussed findings and provided evidence (concrete data) from the pilot study. From the analysis and the interpretation, we identified eight recommendations (or opportunities) to improve the success of priority setting within the organization (Table 8). In an informal debriefing, the senior managers we spoke with (n = 3) all felt useful information was generated that could be translated into positive organizational changes in priority setting. They believed the report captured the essence of the process and that it spoke to the underlying cultural shift in the organization, but that it would be more useful if details on implementing recommendations and on the practices of other hospitals were provided.

**Table 8:** Recommendations from Pilot Study Report

*1. Increase consultation with stakeholders (internal and external) - *the hospital should engage with a broader range of internal and external stakeholders in the budgeting process, including front line staff, other healthcare providers, and the public. This would facilitate buy-in across a range of stakeholder interests, enhances the evidence-base of decisions, and strengthens alignment of decisions with relevant stakeholder values.

*2. Develop an explicit and formalized communication plan - *the hospital should develop a formal communication plan that involves multiple vehicles and includes relevant information on the budgeting process from start to finish. This will enhance the process' transparency -- access to important information, which facilitates more meaningful participation.

*3. Revisit data and information needs - *the decision makers should have access to adequate information and decision support. The hospital should engage with program directors/managers to identify gaps in data and develop strategies for collecting appropriate data.

*4. Include a Revision or Appeals Process - *The hospital should develop a revision process as a constructive way for stakeholders to raise concerns about decisions and to propose reasonable alternatives to improve the quality of decisions.

*5. Improve the computer-based budget tool - *Most participants felt that the computer-based budgeting program was a positive advance that helped the process, but some experienced some frustrations with the programs shortcoming. The hospital should work with participants to improve the program. An improved tool could offer more evidence-based decisions, more confidence in the quality of decisions, and a greater ease in making decisions.

*6. Address Key Timing Concerns - *The hospital should revisit the timing of the budgeting process and reconsider both the length of time and time of year to complete the budgeting in order to allow stakeholders time to review and gather information. This would not only allow for a more explicit decision making process, but could also help create conditions for more effective stakeholder engagement.

*7. Provide Training for Decision Makers - *In order build capacity in decision makers within the organization, the hospital should provide specific training that is tailored to increase the budgeting skill set of decision makers.

*8. Build on lessons learned - *In order to benefit from the lessons learned in this evaluation and to improve future priority setting activities, the hospital should develop improvement strategies based on these recommendations and re-evaluating budgeting process every year, capturing new lessons and improvements with each iteration.

The willingness of the senior management to adopt the recommendations for improvement was further evidence of the usefulness of the evaluation process. As of this writing, the report has resulted in three major changes within the organization including: (1) a change to timing (recommendation #6): the following budgeting process began earlier and not run into the summer; (2) increased information (recommendation #3): the hospital started using a new information database to provide decision makers more with up-to-date and accurate information; and (3) increased stakeholder involvement and training (recommendation #1 and #7): focus group consultations were done in order to learn more about the strengths and weaknesses of the budgeting process and ways the support services can help.

From the researchers' perspective, the pilot test allowed us to gain a better understanding of how the evaluation process functioned in a real-world setting, as well as its applicability in the healthcare context. We were able to evaluate the hospital against the 10 elements of successful priority setting derived from the conceptual framework. We found there were both advantages and disadvantages to being external researchers conducting the assessment. For example, as researchers our expertise was valued and we were not perceived as a threat. On the other hand, we lacked important 'insider' (cultural) knowledge that would have helped to contextualize our findings. The evaluation process was limited in its ability to analyze the budget: the provincial ministry of health has very specific accounting and reporting regulations that hospitals must follow, and regulations can (and do) change from year to year making it difficult to track organizational changes or shifts. Many decisions in reallocation were a direct result of Ministry directives to change protocol for financial records as opposed to deliberate resource shifting decisions.

##### Changes to Conceptual Framework and Evaluation Process

The pilot test provided an opportunity to re-examine the conceptual framework and the evaluation process, as a result, both were revised. Changes were made as a result of: (1) experiences with pilot test/evaluation process implementation; (2) direct comments from interviewees (both from the evaluation process and the debriefing); (3) experiences/results from data analysis; (4) further collaborative conceptual thinking (i.e. interdisciplinary analysis), or (5) a combination of the aforementioned.

In general, the pilot test provided support for our definition of success, (i.e., our conceptual framework), that is, we found the framework both applicable to the context and able to capture the relevant aspects of the budgeting process. Refinements to the conceptual framework focused primarily on wording; for example element labels were changed or refined (Table 9). Other changes were intended to simplify the language for ease of understanding/use, for example, 'Shifted Priorities/Reallocation of Resources' became 'Shifted Resources'. The original definitions of the elements (Table 2) did not change. Further exploration is warranted to explore the applicability of the conceptual framework and the definition of success in other contexts.

**Table 9:** Changes/Refinements to Conceptual Framework

	Elements	Change
**Process**	Stakeholder Engagement	no change

	Use Of Explicit Process	Removed words to simplify
	
	Clear And Transparent Information Management	no change
	
	Consideration Of Values and Context	no change
	
	Revision Or Appeals Mechanism	no change

**Outcomes**	Improved Stakeholder Understanding	Removed 'improved' - - implies a time lapse
	
	Shifted Priorities /Reallocation Of Resources	Removed words to simplify
	
	Improved Decision Making Quality	Removed 'improved' - - implies a time lapse
	
	Stakeholder Acceptance & Satisfaction	no change
	
	Positive Externalities	no change

The pilot test also provided an opportunity to refine the evaluation process such as re-wording questions, and eliminating duplicate information/redundancy. We reduced the questions in the survey from 35 to 26 (with the 26^th ^question to capture demographic information), with the goal of increasing response rates (updated survey can be found in additional file #3). Questions were eliminated to if they were too repetitive, too complicated (poorly designed ranking lists for example) or if they did not generate useful or novel information. For increased organization and ease of use, we added headers to the document analysis portion of the evaluation process.

## Discussion

This research fills a gap in knowledge by developing an evaluation process that can be used by healthcare managers to evaluate and improve priority setting. The strengths of this evaluation process is that it is understandable and concrete, and can help identify good practices as well as opportunities for improvement. The purpose of the evaluation process is to provide a simple, practical way for an organization to evaluate what it means to achieve success in its priority setting activities and identify areas for improvement. This research is complementary to previous studies that identified pieces of successful priority setting (for example [2,23,33], and it builds and expands upon these previous works by describing a broad range of stakeholders' views about successful priority setting and synthesizes them into one conceptual framework, then operationalized them into an evaluation process that can be used by decision makers to improve priority setting.

Given that we lack consensus on the meaning of successful priority setting, the evaluation process we have created is an initial attempt to evaluate priority setting decisions in a specific context. Outcome measures (such as incidence of complications or patient outcomes) can be helpful in evaluating the success of a health care organization, but they do not provide a complete picture of successful priority setting. The evaluation process and refined conceptual framework, presented in this paper, provide a coherent and detailed definition of success. This is the first attempt to create an evaluation process to evaluate the achievement of success in priority setting in health care organizations. The combination of the conceptual framework and the evaluation process provide a definition to the previously vague notion of successful priority setting.

Some might criticize the conceptual framework for not including health outcomes (this point is further addressed in our first paper [13]) and also criticize the evaluation process for the same reason. However our choice to focus narrowly on priority setting success arose from the need to both define and evaluate a successful priority setting intervention - which the decision makers in our empirical work felt was related to priority setting outcomes (such as improved stakeholder understanding, shifted priorities, improved decision making, stakeholder acceptance, and positive externalities). We acknowledge that not everyone will agree with this and some may argue a framework or tool without patient outcomes is incomplete and lacks comprehensiveness. Future applications of the evaluation process may find a gap in this area, indicating an association between health outcomes and priority setting outcomes.

Overall our experience with the evaluation process was very positive. The survey, interview guide, and document analysis were easy to implement and the knowledge generated from them was felt to be a helpful contribution to improving priority setting efforts. The evaluation process proved to be applicable to the budget setting process and healthcare context, as demonstrated by our ability to capture relevant data (describing the process, decision making inputs, etc). From the application of the evaluation process in the hospital we were able to understand and describe the priority setting context, and provide a report (including recommendations for improvement) to the hospital.

While the framework presents unifying ideas that underlie successful priority setting, we anticipate that the evaluation process will need to be modified for use in other contexts. As the evaluation process is applied in more health care organizations or applied repeatedly in an organization, it may become more streamlined, omitting questions that do not provide fruitful or pertinent information. Future research is required to determine the best combination of the components; for example, fewer one-on-one interviews may be needed while the use of surveys could increase.

### Implications for Policy and Practice

This evaluation process can help build capacity in healthcare managers involved in priority setting which has been shown to be an area most in need of improvement [34]. Through the use of the conceptual framework and the evaluation process, organizational leaders can develop a greater capability to incorporate relevant reasons for decision making which is a key element of legitimate and fair priority setting [33]. In addition, the conceptual framework and the evaluation process provide an explicit structure to facilitate organizational learning and innovation. The conceptual framework and evaluation process can ultimately foster a learning environment among all staff - identifying good practices and opportunities for improvement, strategies for good decision-making and organizational involvement throughout the process.

### Further Research

The evaluation process presents ideas that underlie successful priority setting. However, the evaluation process is not intended to be a blueprint for priority setting practices; it is expected that the evaluation process will need to be adjusted for each organization's unique context (add or remove questions). Moreover, as the survey, interview guide, and document analysis guide are applied in more and varied healthcare organizations, it will become more streamlined and efficient.

Future research can help determine the best way to implement this evaluation process. Our pilot test showed that it is possible for an individual external to the organization to implement the survey, interview guide and document analysis - however, this is not ideal; steps need to be taken to make the evaluation process more user-friendly. For example, the specific components of the evaluation process need to be easily linked to the conceptual framework (additional file 3 provides this linking for the new survey). Ideally, as the evaluation becomes more streamlined (with multiple applications, feedback and revision) there will be less need for the interview - allowing the evaluation process to be more cost and time-efficient and user friendly.

Two issues that are specific to the evaluation process and remain unanswered by this study are, first timing of evaluation process implementation (i.e., right after the priority setting, or six month after, or another interval) and second what priority setting process this evaluation process is best suited for (operational versus strategic planning).

By implementing the evaluation process in other organizations in different healthcare contexts, we could compare lessons between hospitals and understand the problems faced in different hospital contexts. Specifically we could:

• Capture lessons from priority setting experiences that could be used to improve future priority setting processes [35];

• Bring that learning to academic literature, in which hospital priority setting is under described, and in particular to provide leadership in the form of 'good' practices that can be shared with other health care organizations; and

• Cultivate learning organizations.

In future research, as the evaluation process is implemented in more organizations, a set of industry 'best practices' or specific solutions could be developed and added to the evaluation process to enhance its helpfulness to hospitals and other healthcare organizations; however it would require constant updating [36].

## Conclusion

The findings from our pilot evaluation are encouraging but there is still much work to be done to refine the evaluation process and, ultimately, improve the quality of priority setting in specific contexts. The evaluation process needs to become more user friendly, allowing healthcare workers without training or expertise in research to use each of the three components. Using this evaluation process, an organization will be able to identify areas of good practice, areas needing improvement, and establish good priority setting practices within healthcare regions.

## Competing interests

The authors declare that they have no competing interests.

## Authors' contributions

SLS was the primary analyst and principal author of the manuscript. DKM conceived the research; both DKM and JLG were involved in data collection and analysis, and were co-authors of the manuscript. RU and PAS were involved in study conception, analysis and drafting the manuscript. All authors read and approved the final manuscript.

## Pre-publication history

The pre-publication history for this paper can be accessed here:

http://www.biomedcentral.com/1472-6963/10/131/prepub

## Supplementary Material

Additional file 1 **Evaluation Tool**. This file contains the complete original version of the evaluation tool that was piloted in the hospital. Click here for file

Additional file 2 **Survey Results**. This file contains a summary of the quantitative survey results for the survey that was administered in the pilot study. For open-ended questions, responses are not included; only a tally of how many participants responded. Click here for file

Additional file 3 **Complete Version of the Tool**. This file contains the complete tool (survey, interview guide and document analysis with all of the post-pilot study changes. Click here for file
