# Supplementary material for: Evaluating priority setting success in healthcare: a pilot study
Source: BMC Health Serv Res. 2010 May 19;10:131. doi: 10.1186/1472-6963-10-131 (PMC2890637; doi:10.1186/1472-6963-10-131)
Supplement: Additional file 1 — Evaluation Tool. This file contains the complete original version of the evaluation tool that was piloted in the hospital. [file 1472-6963-10-131-S1.DOC]

# APPENDIX

# COMPLETE RESULTS OF THE SURVEY

| **Q1. Were you aware of process and steps involved in the 2007-2008 budgeting?** | | | | | | |  |  |
| --- | --- | --- | --- | --- | --- | --- | --- | --- |
|  |  |  |  |  |  |  |  |  |
| *Answer Options* | **n** | *%* |  |  |  |  |  |  |
| Yes | 38 | 36.20% |  |  |  |  |  |  |
| No | 62 | 59.10% |  |  |  |  |  |  |
| I don't know | 5 | 4.80% |  |  |  |  |  |  |
| *Total* | *105* |  |  |  |  |  |  |  |
| *(skipped)* | *0* |  |  |  |  |  |  |  |
|  |  |  |  |  |  |  |  |  |
| **Q2. Do you think the process was fair?** | | |  |  |  |  |  |  |
|  |  |  |  |  |  |  |  |  |
| *Answer Options* | **n** | **%** |  |  |  |  |  |  |
| yes | 21 | 20.00% |  |  |  |  |  |  |
| no | 11 | 10.50% |  |  |  |  |  |  |
| I don't know | 73 | 69.50% |  |  |  |  |  |  |
| *Total* | 105 |  |  |  |  |  |  |  |
| *(skipped)* | 0 |  |  |  |  |  |  |  |
|  |  |  |  |  |  |  |  |  |
| **Q3. Did you understand the purpose and goals of the 2007-2008 budgeting process?** | | | | | | |  |  |
|  |  |  |  |  |  |  |  |  |
| *Answer Options* | **n** | **%** |  |  |  |  |  |  |
| Yes | 47 | 45.20% |  |  |  |  |  |  |
| No | 57 | 54.80% |  |  |  |  |  |  |
| *Total* | 104 |  |  |  |  |  |  |  |
| *(skipped)* | 1 |  |  |  |  |  |  |  |
|  |  |  |  |  |  |  |  |  |
| **Q4. Was there an explicit and predetermined timeline for the 2007-2008 budgeting process?** | | | | | | | |  |
|  |  |  |  |  |  |  |  |  |
| *Answer Options* | **n** | **%** |  |  |  |  |  |  |
| Yes | 44 | 42.30% |  |  |  |  |  |  |
| No | 4 | 3.90% |  |  |  |  |  |  |
| I don't know | 56 | 53.90% |  |  |  |  |  |  |
| *Total* | 104 |  |  |  |  |  |  |  |
| *(skipped)* | 1 |  |  |  |  |  |  |  |
|  |  |  |  |  |  |  |  |  |
| **Q5. In comparison to previous decision making or priority setting at [the hospital], is there consistency in reasoning between previous and the 2007-2008 budgeting process?** | | | | | | | | |
|  |  |  |  |  |  |  |  |  |
| *Answer Options* | **n** | **%** |  |  |  |  |  |  |
| Yes | 10 | 9.70% |  |  |  |  |  |  |
| No | 17 | 16.50% |  |  |  |  |  |  |
| I don't know | 76 | 73.80% |  |  |  |  |  |  |
| *Total* | 103 |  |  |  |  |  |  |  |
| *(skipped)* | 2 |  |  |  |  |  |  |  |
|  |  |  |  |  |  |  |  |  |
|  |  |  |  |  |  |  |  |  |
| **Q6. Was there a revision or appeals process available (whereby a decision could be contested or reviewed)?** | | | | | | | | |
|  |  |  |  |  |  |  |  |  |
| *Answer Options* | **n** | **%** |  |  |  |  |  |  |
| Yes | 8 | 9.60% |  |  |  |  |  |  |
| No | 17 | 20.50% |  |  |  |  |  |  |
| I don't know | 58 | 69.90% |  |  |  |  |  |  |
| *Total* | 83 |  |  |  |  |  |  |  |
| *(skipped)* | 22 |  |  |  |  |  |  |  |
|  |  |  |  |  |  |  |  |  |
| **Q7. Was there integration of [the hospital]'s 2007-2008 budgeting process with other healthcare organizations?** | | | | | | | | |
|  |  |  |  |  |  |  |  |  |
| *Answer Options* | **n** | **%** |  |  |  |  |  |  |
| Yes | 8 | 9.60% |  |  |  |  |  |  |
| No | 10 | 12.10% |  |  |  |  |  |  |
| I don't know | 65 | 78.30% |  |  |  |  |  |  |
| *Total* | 83 |  |  |  |  |  |  |  |
| *(skipped)* | 22 |  |  |  |  |  |  |  |
|  |  |  |  |  |  |  |  |  |
| **Q8. During the 2007-2008 budgeting process, the following were considered:** | | | | | |  |  |  |
|  |  |  |  |  |  |  |  |  |
| *Answer Options* | **n** | strongAgree | agree | disagree | strongDisagree | N/A |  |  |
| [the hospital]'s Mission, Vision, and Values | 70 | 8 | 34 | 12 | 2 | 14 |  |  |
| [the hospital] Strategic Plan | 70 | 8 | 38 | 9 | 1 | 14 |  |  |
| [the hospital] Context | 68 | 7 | 32 | 6 | 2 | 21 |  |  |
| [the hospital] Culture | 68 | 5 | 30 | 10 | 4 | 19 |  |  |
| Community Values | 68 | 3 | 33 | 13 | 3 | 16 |  |  |
| Patient Values | 68 | 5 | 29 | 17 | 2 | 15 |  |  |
| Staff Values | 68 | 4 | 19 | 21 | 6 | 18 |  |  |
| *Total* | 70 |  |  |  |  |  |  |  |
| *(skipped)* | 35 |  |  |  |  |  |  |  |
|  |  |  |  |  |  |  |  |  |
| **Q9. Are there other items that should have been considered in the 2007-2008 budgeting process?** | | | | | | | |  |
|  |  |  |  |  |  |  |  |  |
| *Answer Options* | **n** | **%** |  |  |  |  |  |  |
| No | 23 | 39.00% |  |  |  |  |  |  |
| Yes; please specify | 36 | 61.00% |  |  |  |  |  |  |
| *Total* | 59 |  |  |  |  |  |  |  |
| *(skipped)* | 46 |  |  |  |  |  |  |  |
|  |  |  |  |  |  |  |  |  |
| **Q10. Are there items that were considered in the 2007-2008 budgeting process that should NOT have been?** | | | | | | | | |
|  |  |  |  |  |  |  |  |  |
| *Answer Options* | **n** | **%** |  |  |  |  |  |  |
| No | 42 | 80.80% |  |  |  |  |  |  |
| Yes; please specify | 10 | 19.20% |  |  |  |  |  |  |
| *Total* | 52 |  |  |  |  |  |  |  |
| *(skipped)* | 53 |  |  |  |  |  |  |  |
|  |  |  |  |  |  |  |  |  |
| **Q11. Were there multiple methods of engaging stakeholders/decision makers?** | | | | | |  |  |  |
|  |  |  |  |  |  |  |  |  |
| *Answer Options* | **n** | **%** |  |  |  |  |  |  |
| Yes | 12 | 15.40% |  |  |  |  |  |  |
| No | 13 | 16.70% |  |  |  |  |  |  |
| I don't know | 53 | 68.00% |  |  |  |  |  |  |
| *Total* | 78 |  |  |  |  |  |  |  |
| *(skipped)* | 27 |  |  |  |  |  |  |  |
|  |  |  |  |  |  |  |  |  |
| **Q12. Were these methods effective?** | |  |  |  |  |  |  |  |
|  |  |  |  |  |  |  |  |  |
| *Answer Options* | **n** | **%** |  |  |  |  |  |  |
| yes | 5 | 7.30% |  |  |  |  |  |  |
| no | 14 | 20.30% |  |  |  |  |  |  |
| I don't know | 50 | 72.50% |  |  |  |  |  |  |
| *Total* | 69 |  |  |  |  |  |  |  |
| *(skipped)* | 36 |  |  |  |  |  |  |  |
|  |  |  |  |  |  |  |  |  |
| **Q13. Please explain.(Open-Ended)** | |  |  |  |  |  |  |  |
| *Total* | 18 |  |  |  |  |  |  |  |
| *(skipped)* | 87 |  |  |  |  |  |  |  |
|  |  |  |  |  |  |  |  |  |
| **Q14. Was everyone involved in the 2007-2008 budget who should have been?** | | | | | |  |  |  |
|  |  |  |  |  |  |  |  |  |
| *Answer Options* | **n** | **%** |  |  |  |  |  |  |
| Yes | 9 | 11.70% |  |  |  |  |  |  |
| I don't know | 48 | 62.30% |  |  |  |  |  |  |
| No | 20 | 26.00% |  |  |  |  |  |  |
| *Total* | 77 | *answered* |  |  |  |  |  |  |
| *(skipped)* | 28 | *skipped* |  |  |  |  |  |  |
|  |  |  |  |  |  |  |  |  |
| **Q15. How involved in the 2007-2008 budget were you?** | | | |  |  |  |  |  |
|  |  |  |  |  |  |  |  |  |
| *Answer Options* | **n** | **%** |  |  |  |  |  |  |
| Very involved | 17 | 21.80% |  |  |  |  |  |  |
| Somewhat involved | 15 | 19.20% |  |  |  |  |  |  |
| Not at all involved | 46 | 59.00% |  |  |  |  |  |  |
| *Total* | 78 |  |  |  |  |  |  |  |
| *(skipped)* | 27 |  |  |  |  |  |  |  |
|  |  |  |  |  |  |  |  |  |
| **Q16. Were you satisfied with your involvement in the 2007-2008 budget?** | | | | | |  |  |  |
|  |  |  |  |  |  |  |  |  |
| *Answer Options* | **n** | **%** |  |  |  |  |  |  |
| Yes | 20 | 26.70% |  |  |  |  |  |  |
| No | 28 | 37.30% |  |  |  |  |  |  |
| Not Sure | 27 | 36.00% |  |  |  |  |  |  |
| *Total* | 75 |  |  |  |  |  |  |  |
| *(skipped)* | 30 |  |  |  |  |  |  |  |
|  |  |  |  |  |  |  |  |  |
| **Q17. Please explain. (Open-Ended)** | |  |  |  |  |  |  |  |
| *answered* | 21 |  |  |  |  |  |  |  |
| *skipped* | 84 |  |  |  |  |  |  |  |
|  |  |  |  |  |  |  |  |  |
| **Q18. Do you know how the decisions for the 2007-2008 budget were made?** | | | | | |  |  |  |
|  |  |  |  |  |  |  |  |  |
| *Answer Options* | **n** | **%** |  |  |  |  |  |  |
| Yes | 13 | 16.70% |  |  |  |  |  |  |
| No | 39 | 50.00% |  |  |  |  |  |  |
| Not Sure | 26 | 33.30% |  |  |  |  |  |  |
| *Total* | 78 |  |  |  |  |  |  |  |
| *(skipped)* | 27 |  |  |  |  |  |  |  |
|  |  |  |  |  |  |  |  |  |
| **Q19. Do you know who was making the decisions for the 2007-2008 budget?** | | | | | |  |  |  |
|  |  |  |  |  |  |  |  |  |
| *Answer Options* | **n** | **%** |  |  |  |  |  |  |
| No | 22 | 28.20% |  |  |  |  |  |  |
| I don't know | 27 | 34.60% |  |  |  |  |  |  |
| Yes; Please state who | 29 | 37.20% |  |  |  |  |  |  |
| *Total* | 78 |  |  |  |  |  |  |  |
| *(skipped)* | 27 |  |  |  |  |  |  |  |
|  |  |  |  |  |  |  |  |  |
| **Q20. For the following elements of the 2007-2008 budget, please if they were communicated to you.** | | | | | | | |  |
|  |  |  |  |  |  |  |  |  |
| *Answer Options* | **n** | yes | no | I don't know | N/A |  |  |  |
| Purpose & Goals of Process | 71 | 35 | 24 | 9 | 3 |  |  |  |
| Methods | 70 | 29 | 27 | 11 | 3 |  |  |  |
| Outcomes | 71 | 29 | 29 | 10 | 3 |  |  |  |
| Revision/Appeals Process | 71 | 8 | 44 | 16 | 3 |  |  |  |
| *Total* | 71 |  |  |  |  |  |  |  |
| *(skipped)* | 34 |  |  |  |  |  |  |  |
|  |  |  |  |  |  |  |  |  |
| **Q21. For the following elements, please indicate how well they were communicated to you.** | | | | | | | |  |
|  |  |  |  |  |  |  |  |  |
| *Answer Options* | **n** | were not | very poorly | adequately | well | very well | N/A |  |
| Purpose & Goals | 67 | 20 | 9 | 26 | 6 | 2 | 4 |  |
| Methods | 67 | 23 | 11 | 19 | 6 | 1 | 7 |  |
| Outcomes | 66 | 21 | 15 | 17 | 6 | 1 | 6 |  |
| Revision/Appeals Process | 67 | 37 | 11 | 7 | 3 | 1 | 8 |  |
| *Total* | 68 |  |  |  |  |  |  |  |
| *(skipped)* | 37 |  |  |  |  |  |  |  |
|  |  |  |  |  |  |  |  |  |
| **Q22. How were the above items communicated to you? (check all that apply)** | | | | | |  |  |  |
|  |  |  |  |  |  |  |  |  |
| *Answer Options* | **n** |  |  |  |  |  |  |  |
| E-mail | 28 |  |  |  |  |  |  |  |
| Paystub | 0 |  |  |  |  |  |  |  |
| Hospital Newsletter | 2 |  |  |  |  |  |  |  |
| Announcement Posting | 4 |  |  |  |  |  |  |  |
| Meeting: Departmental | 26 |  |  |  |  |  |  |  |
| Meeting: Hospital-Wide | 18 |  |  |  |  |  |  |  |
| Peer-to-Peer Informal | 21 |  |  |  |  |  |  |  |
| Peer-to-Peer Formal | 16 |  |  |  |  |  |  |  |
| *Total* | 48 |  |  |  |  |  |  |  |
| *(skipped)* | 57 |  |  |  |  |  |  |  |
|  |  |  |  |  |  |  |  |  |
| **Q23. Other methods of communication. (Open-Ended)** | | | |  |  |  |  |  |
| *answered* | 11 |  |  |  |  |  |  |  |
| *skipped* | 94 |  |  |  |  |  |  |  |
|  |  |  |  |  |  |  |  |  |
|  |  |  |  |  |  |  |  |  |
| **Q24. How could communication be improved? (Open-Ended)** | | | | |  |  |  |  |
| *answered* | 31 |  |  |  |  |  |  |  |
| *skipped* | 74 |  |  |  |  |  |  |  |
|  |  |  |  |  |  |  |  |  |
| **Q25. Do you understand the outcome of the 2007-2008 budget?** | | | | |  |  |  |  |
|  |  |  |  |  |  |  |  |  |
| *Answer Options* | **n** | **%** |  |  |  |  |  |  |
| Yes, completely understand | 11 | 15.90% |  |  |  |  |  |  |
| Somewhat understand | 32 | 46.40% |  |  |  |  |  |  |
| No, don't understand | 26 | 37.70% |  |  |  |  |  |  |
| *Total* | 69 |  |  |  |  |  |  |  |
| *(skipped)* | 36 |  |  |  |  |  |  |  |
|  |  |  |  |  |  |  |  |  |
| **Q26. Do you accept the outcomes of the 2007-2008 budget?** | | | | |  |  |  |  |
|  |  |  |  |  |  |  |  |  |
| *Answer Options* | **n** | **%** |  |  |  |  |  |  |
| Yes, completely accept | 9 | 12.90% |  |  |  |  |  |  |
| Somewhat accept | 29 | 41.40% |  |  |  |  |  |  |
| No, don't at all accept | 3 | 4.30% |  |  |  |  |  |  |
| I don't know | 29 | 41.40% |  |  |  |  |  |  |
| *Total* | 70 |  |  |  |  |  |  |  |
| *(skipped)* | 35 |  |  |  |  |  |  |  |
|  |  |  |  |  |  |  |  |  |
| **Q27. Are you satisfied with the outcomes of the 2007-2008 budget?** | | | | |  |  |  |  |
|  |  |  |  |  |  |  |  |  |
| *Answer Options* | **n** | **%** |  |  |  |  |  |  |
| Yes, completely satisfied | 5 | 7.10% |  |  |  |  |  |  |
| Somewhat satisfied | 22 | 31.40% |  |  |  |  |  |  |
| No, not satisfied | 16 | 22.90% |  |  |  |  |  |  |
| I don't know | 27 | 38.60% |  |  |  |  |  |  |
| *Total* | 70 |  |  |  |  |  |  |  |
| *(skipped)* | 35 |  |  |  |  |  |  |  |
|  |  |  |  |  |  |  |  |  |
| **Q28. Now that the 2007-2008 budgeting process is finished, please indicate if you are more familiar with the following items** | | | | | | | | |
|  |  |  |  |  |  |  |  |  |
| *Answer Options* | **n** | more | no, not more | same as before | I don't know |  |  |  |
| [the hospital] Mission, vision, values | 61 | 5 | 14 | 37 | 5 |  |  |  |
| [the hospital] Strategic plan | 62 | 8 | 16 | 32 | 6 |  |  |  |
| [the hospital] Context | 56 | 1 | 19 | 28 | 8 |  |  |  |
| [the hospital] Culture | 55 | 2 | 16 | 30 | 7 |  |  |  |
| Community Values | 55 | 4 | 18 | 28 | 5 |  |  |  |
| Patient Values | 55 | 3 | 18 | 29 | 5 |  |  |  |
| Staff Values | 58 | 5 | 18 | 30 | 5 |  |  |  |
| *Total* | 67 |  |  |  |  |  |  |  |
| *(skipped)* | 38 |  |  |  |  |  |  |  |
|  |  |  |  |  |  |  |  |  |
| **Q29. To what degree are the following items reflected in the 2007-2008 budget?** | | | | | | |  |  |
|  |  |  |  |  |  |  |  |  |
| *Answer Options* | **n** | not | somewhat | appropriately | very | overly |  |  |
| [the hospital] Mission, Vision,& Values | 42 | 4 | 14 | 22 | 2 | 0 |  |  |
| [the hospital] Strategic Plan | 41 | 1 | 15 | 21 | 4 | 0 |  |  |
| [the hospital] Context | 39 | 5 | 15 | 16 | 3 | 0 |  |  |
| [the hospital] Culture | 39 | 8 | 15 | 15 | 1 | 0 |  |  |
| Community Values | 40 | 10 | 15 | 15 | 0 | 0 |  |  |
| Patient Values | 38 | 9 | 14 | 14 | 1 | 0 |  |  |
| Staff Values | 39 | 13 | 13 | 12 | 1 | 0 |  |  |
| *Total* | 42 |  |  |  |  |  |  |  |
| *(skipped)* | 63 |  |  |  |  |  |  |  |
|  |  |  |  |  |  |  |  |  |
| **Q30. How satisfied were you with the process behind the 2007-2008 budget?** | | | | | |  |  |  |
|  |  |  |  |  |  |  |  |  |
| *Answer Options* | **n** | **%** |  |  |  |  |  |  |
| Not at all satisfied | 18 | 28.10% |  |  |  |  |  |  |
| Somewhat satisfied | 13 | 20.30% |  |  |  |  |  |  |
| Neutral | 27 | 42.20% |  |  |  |  |  |  |
| Satisfied | 5 | 7.80% |  |  |  |  |  |  |
| Very satisfied | 1 | 1.60% |  |  |  |  |  |  |
| *Total* | 64 |  |  |  |  |  |  |  |
| *(skipped)* | 41 |  |  |  |  |  |  |  |
|  |  |  |  |  |  |  |  |  |
| **Q31. Please explain. (Open-Ended)** | |  |  |  |  |  |  |  |
| *answered* | 26 |  |  |  |  |  |  |  |
| *skipped* | 79 |  |  |  |  |  |  |  |
|  |  |  |  |  |  |  |  |  |
| **Q32. How satisfied were you with the outcomes of the 2007-2008 budget?** | | | | | |  |  |  |
|  |  |  |  |  |  |  |  |  |
| *Answer Options* | **n** | **%** |  |  |  |  |  |  |
| Not at all satisfied | 16 | 25.00% |  |  |  |  |  |  |
| Somewhat satisfied | 12 | 18.80% |  |  |  |  |  |  |
| Neutral | 29 | 45.30% |  |  |  |  |  |  |
| Satisfied | 6 | 9.40% |  |  |  |  |  |  |
| Very satisfied | 1 | 1.60% |  |  |  |  |  |  |
| *Total* | 64 |  |  |  |  |  |  |  |
| *(skipped)* | 41 |  |  |  |  |  |  |  |
|  |  |  |  |  |  |  |  |  |
| **Q33. Please explain.(Open-Ended)** | |  |  |  |  |  |  |  |
| *answered* | 21 |  |  |  |  |  |  |  |
| *skipped* | 84 |  |  |  |  |  |  |  |
|  |  |  |  |  |  |  |  |  |
| **Q34. How would you improve/what changes would you make to the 2007-2008 budgeting process? (Open-Ended)** | | | | | | |  |  |
| *answered* | 28 |  |  |  |  |  |  |  |
| *skipped* | 77 |  |  |  |  |  |  |  |
