# Supplementary material for: Evaluating priority setting success in healthcare: a pilot study
Source: BMC Health Serv Res. 2010 May 19;10:131. doi: 10.1186/1472-6963-10-131 (PMC2890637; doi:10.1186/1472-6963-10-131)
Supplement: Additional file 3 — Complete Version of the Tool. This file contains the complete tool (survey, interview guide and document analysis with all of the post-pilot study changes. [file 1472-6963-10-131-S3.DOC]

# Additional File #3:

# COMPLETE VERSION OF TOOL (POST-PILOT TEST CHANGES)

**SURVEY**

1. Were you aware of that [THE HEALTHCARE ORGANIZATION] had a priority setting process? (yes/no)
2. How involved were you in the priority setting? (very involved, somewhat involved, not at all involved)
3. Were you satisfied with your involvement in the priority setting process? (yes/no/not sure)

**Information and Communication**

1. For the following elements of the priority setting process, please if they were communicated to you. (yes/no/I don’t know)
2. Purpose & Goals
3. Methods
4. Outcomes
5. Revision/Appeals
6. For the following elements of the priority setting process, please indicate how well they were communicated to you. (rank: they were not, very poorly, adequately, well, very well, and N/A)
7. Purpose & Goals
8. Methods
9. Outcomes
10. Revision/Appeals
11. Did you understand the purpose and goals of the priority setting process? (yes/no)
12. How could the communication be improved? (open-ended)

**Process**

1. Was there an explicit and predetermined timeline for the priority setting process? (yes/no/I don’t know)
2. Was there a revision or appeals process available (whereby a decision could be contested or reviewed)? (yes/no/I don’t know)
3. During the priority setting process, the following items were considered: (not considered and that is ok, not considered but should have been, considered the appropriate amount, considered but should be considered more, considered too much)
4. [THE HEALTHCARE ORGANIZATION] Mission, vision, values
5. [THE HEALTHCARE ORGANIZATION] Strategic plan
6. [THE HEALTHCARE ORGANIZATION] Context
7. [THE HEALTHCARE ORGANIZATION] Culture
8. Community Values
9. Patient Values
10. Staff Values
11. Are there other items that should have been considered in the priority setting process? (yes/no and please specify)

**Stakeholder Engagement**

1. Were there multiple methods of engaging stakeholders/decision makers? (yes/no/I don’t know)
2. Were these methods successful? (yes/no/I don’t know)
3. Do you know how the decisions for the priority setting process were made? (yes/no/I don’t know)
4. Do you know who was making the decisions for the priority setting process? (yes/no/I don’t know and please state who)
5. Who should have been involved in the priority setting process that was not? (skip question or open-ended)

**Outcomes**

1. Do you understand the outcome of the priority setting? (yes, completely understand; somewhat understand; no, don’t understand)
2. Do you accept the outcomes of the priority setting? (yes, completely accept; somewhat accept; no, don’t accept)
3. Are you satisfied with the outcomes of the priority setting? (yes, completely satisfied; somewhat satisfied; no, not satisfied)
4. To what degree are the following items reflected in the priority setting? (not reflected and that is ok, not reflected but should have been, reflected the appropriate amount, reflected but should be considered more, reflected too much)
5. [THE HEALTHCARE ORGANIZATION] Mission, vision, values
6. [THE HEALTHCARE ORGANIZATION] Strategic plan
7. [THE HEALTHCARE ORGANIZATION] Context
8. [THE HEALTHCARE ORGANIZATION] Culture
9. Community Values
10. Patient Values
11. Staff Values
12. In comparison to previous decision making or priority setting at [THE HEALTHCARE ORGANIZATION], is there consistency in reasoning between previous and the priority setting process? (yes/no/I don’t know)
13. Was there integration of [THE HEALTHCARE ORGANIZATION]'s priority setting process with other healthcare organizations? (yes/no/I don’t know)

**Overall View of the Process** – these are the last three formal questions of the survey.

1. Do you think the process was fair? (yes/no/I don’t know)
2. How satisfied were you with the process behind the priority setting? (Completely satisfied, somewhat satisfied, not at all satisfied)
3. How would you improve/what changes would you make to the priority setting process? (open-ended)

**INTERVIEW GUIDE**

1. Please talk to me about who was involved in the priority setting process and how they were involved.
2. Tell me about the priority setting process.
   - 1. Was there an explicit & transparent process?
     2. What were the major considerations? (values, culture, context)
3. What happened if people did not agree with the decisions or the process?
4. How are things different from before this priority setting process?
5. How were the decisions reflected elsewhere in the organization?
6. What did you learn from the priority setting process?
7. Improved knowledge or understanding of the organization? (e.g. strategic plan; mission, vision and values; staff/community values)
8. How would you improve the priority setting process?
9. How satisfied were you with the priority setting process overall?

**DOCUMENT ANALYSIS**

Analyze Communication Documents: (meeting minutes, emails, memos, website, etc)

1. Is there a record of who was involved during each phase of the priority setting process? Are the records consistent?
2. Is there a record of the process by which decisions were made and the people involved?
3. What forms of communication were used?
4. Was there a clear communication plan?
5. Are there any documents surrounding the use of an appeal process? What did the process look like?

Analyze Information/Handouts:

1. Is there a description of the priority setting process in documents?
2. What information/data was used to inform the priority setting process?

Analyze Mission/Vision/Values & Strategic Plan:

1. Were the mission, vision and values considered during the process or changed/revised after the process?

Analyze Budget:

1. Does the budget reflect a change in resources or priorities given to programs?
2. Does the budget have similar or different goals/priorities than other organizational documents (e.g. strategic plan, other departmental/program budgets)?

Analyze External Documents:

1. Were any media reports generated from this priority setting process? (before, during or after?) (Internally or externally driven?)
2. Has there been any legislation or policy changes as a result of this priority setting process?
